# *RAB33B* and *PCNT* variants in two Pakistani families with skeletal dysplasia and short stature

**DOI:** 10.1186/s12891-021-04503-2

**Published:** 2021-07-20

**Authors:** Noor ul Ain, Zunaira Fatima, Sadaf Naz, Outi Makitie

**Affiliations:** 1 School of Biological Sciences, University of the Punjab, Quaid-i-Azam Campus, Lahore, 54590 Pakistan; 2 Department of Molecular Medicine and Surgery and Center for Molecular Medicine, Karolinska Institutet, Stockholm, Sweden; 3 Present address: Institute of Biomedical and Genetic Engineering, Islamabad, Pakistan; 4 Folkhälsan Institute of Genetics, Helsinki, Finland; 5 Children’s Hospital, University of Helsinki and Helsinki University Hospital, P.O. Box 63, 00014 Helsinki, Finland

**Keywords:** Skeletal dysplasia, Short stature, Whole genome sequencing, Smith-McCort dysplasia, MOPDII, Pakistan

## Abstract

**Background:**

Skeletal dysplasia is a heterogeneous group of disorders resulting from different genetic variants in humans. The current study was designed to identify the genetic causes of skeletal dysplasia and short stature in two consanguineous families from Pakistan, both comprised of multiple affected individuals. Patients in one family had proportionate short stature with reduced head circumference while affected individuals in the other family had disproportionate short stature.

**Methods:**

Clinical data were obtained and radiological examinations of the index patients were completed. Whole genome sequencing for probands from both families were performed followed by Sanger sequencing to confirm segregation of identified variants in the respective families. In-silico pathogenicity score prediction for identified variant and amino acid conservation analysis was completed.

**Results:**

Whole Genome Sequencing identified a known biallelic variant c.6176_6189delGTCAGCTGCCGAAG; p.(Gln2060ArgfsTer48) in *PCNT* gene and a novel biallelic variant c.174delC; p.(Asp60ThrfsTer7) in *RAB33B* gene respectively in affected members of the two families. Clinical imaging revealed platyspondyly and varus deformity in the legs of the affected members in the first family. Radiographs indicated severe platyspondyly, genu valgus deformity of legs and pectus carinatum for the patients in the second family.

**Conclusion:**

In this study we report the phenotypes and genetic variants in two unrelated families with two distinct forms of skeletal dysplasia. This study strengthens the previous findings that patients harboring *PCNT* variants are phenotypically homogeneous and also extends the genotypic spectrum of *RAB33B* variants.

**Supplementary Information:**

The online version contains supplementary material available at 10.1186/s12891-021-04503-2.

## Background

Skeletal dysplasias are a heterogeneous group of disorders, each manifesting with variable degrees of skeletal anomalies. In many cases, stunted growth and metabolic abnormalities are also observed. Microcephalic osteodysplastic primordial dwarfism type II and Smith–McCort dysplasia are two distinct autosomal recessive skeletal dysplasias with severe short stature.

Microcephalic osteodysplastic primordial dwarfism type II (MOPDII) (OMIM 210,720) is caused by pathogenic variants of *PCNT* (OMIM 605,925)*,* which are also associated with other human disorders including Seckel syndrome [1]. MOPDII and Seckel syndrome are distinguishable even though they have some common features such as disproportionate short stature and specific skeletal deformities. Pericentrin (PCNT), encoded by *PCNT* gene, is a highly coiled coil protein, with a conserved C terminal region responsible for interaction with other protein components [2].

Variants in *RAB33B* have been identified in patients with Smith–McCort dysplasia (SMC) negative for *DYM* mutations [3, 4]. *RAB33B* belongs to a group in the Rab family which includes small GTP binding proteins. These proteins have a crucial role in cellular trafficking such as exocytosis and endocytosis [3, 4]. Smith–McCort dysplasia (SMC) (OMIM 607,326) and Dyggve–Melchior–Clausen syndrome (DMC) (OMIM 223,800) are two allelic syndromes caused by variants of *DYM* [5]. Both Smith–McCort dysplasia (SMC) and Dyggve–Melchior–Clausen syndrome (DMC) share same distinctive skeletal features such as platyspondyly, abnormalities in epiphyses and metaphyses, and irregular contours of iliac crest along with spinal cord compression. SMC and DMC are distinguishable on the basis of intellectual abilities with cognition being normal in SMC [3].

In the present study we investigated two families with multiple affected individuals exhibiting skeletal dysplasia. We performed whole genome sequencing and identified the pathogenic alleles segregating with the disorders.

## Methods

### Subjects

**Fig. 1:**
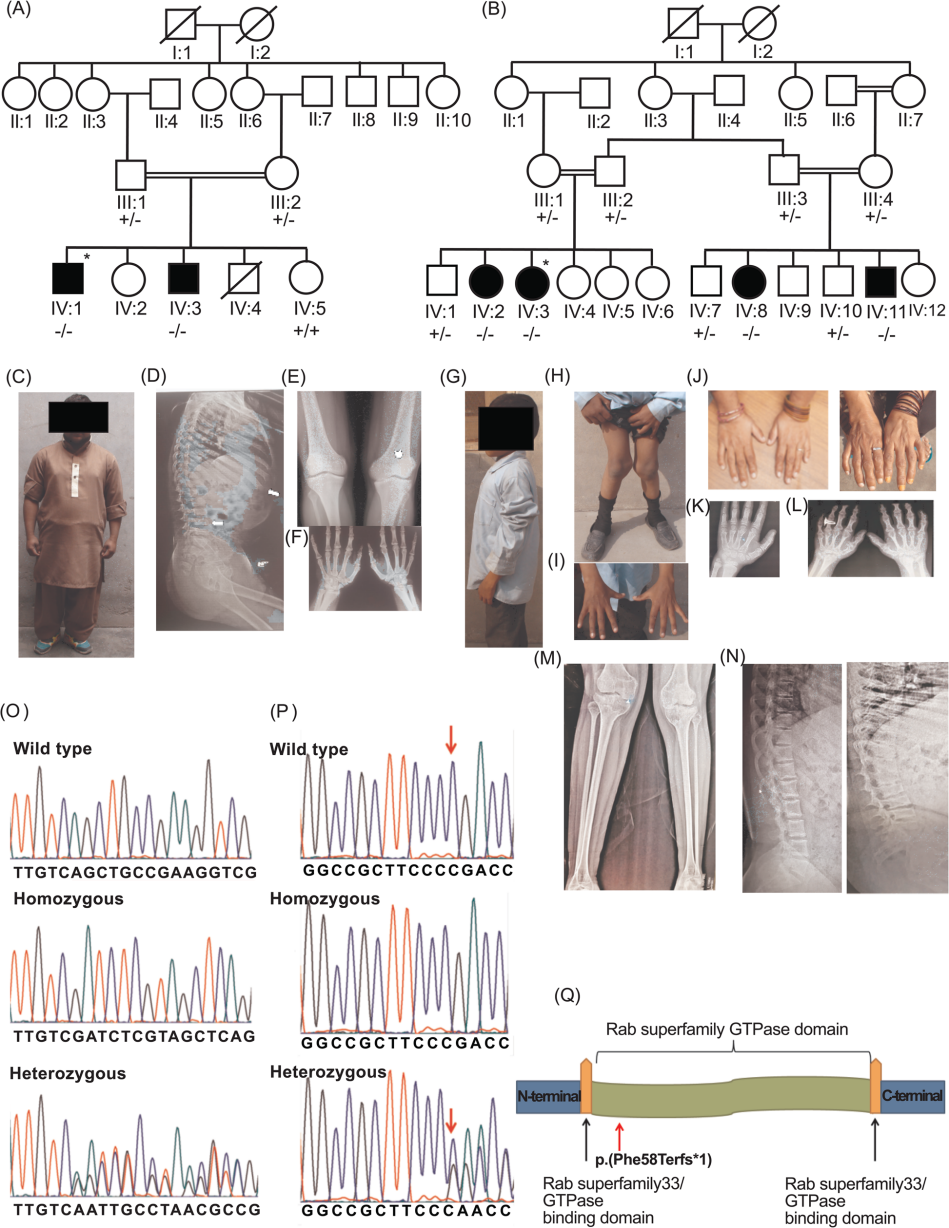
Clinical features of families and segregation of identified variants. **A.** Family pedigree of ZFD-01. Filled symbols indicate the affected individuals. Double lines depict consanguinity. An asterisk (*) indicates the individual for whom whole genome sequencing was performed. Genotypes are provided for the individuals whose samples were collected. **B**. Family pedigree of ZFD-02. **C.** An affected individual IV:3 from family ZFD-01: proportionate short stature is evident. **D**. Lateral view of his spine radiograph shows severe platyspondyly (arrow) and metaphyseal cupping at distal ribs (arrowhead). **E**. Lower limb radiograph shows slightly abnormal metaphyseal shaping (overtubulation) of the distal femurs, thin fibulae (arrow) and mild valgus deformity with laterally placed patellae (arrowhead). **F**. Radiograph of the hands of IV:3 of ZFD-01 revealed short metacarpals and abnormality in the shape of the carpal structures. **G**. An affected individual IV:11 from family ZFD-02. **H.** His legs showing vagus deformity **I.** hands are normal while **J.** the older individuals show signs of arthropathy. **K.** Hand radiographs show normal structures, including normal carpal bones (arrowhead) in the youngest subject **L**. older subject shows significant loss of articular cartilage in the digits and the wrist, together with erosion of the carpal bones. **M**. Radiographs of the lower limbs of the boy (IV:11 of ZFD-02) show gracile bones and severe valgus deformity **N**. The spine radiograph in the boy (left) shows normal vertebral heights but severe platysplondyly in the older individual (right). **O.** Partial chromatograms of *PCNT* sequence **P**. Partial chromatograms of DNA sequence of *RAB33B*. The red arrow indicates the deleted nucleotide. **Q**. A visual representation of RAB33B protein, red arrow indicates the frameshift variant

Two families ZFD-01 and ZFD-02 (Fig. 1A-B) were identified from Shahkot region of Pakistan after study approval by the Institutional Review Board of School of Biological Sciences, University of the Punjab, Lahore. Written informed consents were obtained from the participants and parents of minors. Clinical and family histories were recorded. Radiographs for hand, spine and legs were obtained. DNA was extracted by a standard protocol involving sucrose lysis, salting out and isopropanol precipitation from blood samples of the participants [6]. All procedures were performed in accordance with relevant guidelines.

### Whole genome sequencing

Whole genome sequencing was performed on samples of affected individuals IV:1 of ZFD-01 and IV:3 of ZFD-02 respectively to identify the cause of the disorders. DNA libraries were constructed using the IlluminaTruSeq PCR-free method. Pair-end reads (2 × 150 bp) were obtained by sequencing on the HiSeqX instrument (Illumina) at the SciLifeLab facility, Stockholm, Sweden. The average coverage of reads was 30X and data were processed according to the Clinical Genetics laboratory, Centre of Molecular Medicine, Karolinska Institutet in-house pipeline. Burrow-Wheeler Aligner (BWA) was used for mapping the reads to the human genome (assembly b37); Genome Analysis Toolkit (GATK) was used for duplicate marking, variant calling and joint genotyping. Annotation of variants was performed using Variant Effect Predictor (VEP).Variant prioritization was based on: 1) autosomal recessive pattern of inheritance (based on family history and the known consanguinity in the family), 2) minor allele frequency (MAF) less than 0.01 in the public databases gnomAD as well as SweGen (Karolinska Institutet in house database), and 3) impact severity based on the variant being frameshift, introducing or deleting stop codons, affecting splice sites and missense variants with high pathogenicity scores.

### Sanger sequencing

The shortlisted variants were checked for segregation in each family by Sanger sequencing in all available DNA samples. Primers were designed using Primer3.0 (https://bioinfo.ut.ee/primer3-0.4.0/).

## Results

### Clinical Findings

The two affected individuals in family ZFD-01(IV:1 and IV:3) had proportionate short stature and typical features of MOPDII (Fig. 1C). The heights of the affected individuals, aged 27 and 20 years, were below -6.8 SD (Table 1). The head circumference was below the normal mean for males (58.4 cm), but cognition was not impaired. Their joints and ability to walk were unaffected. Radiographs of individual IV:3 indicated severe platyspondyly in vertebral bodies, along with a slight varus deformity in legs, abnormal metaphyses in distal femurs and slight abnormality in carpal structure (Fig. 1D-F). The phenotypically normal individuals had no skeletal deformities and exhibited normal growth and heights (Table 1).

**Table 1:** Clinical features of individuals of participating families ZFD-01 and ZFD-02

Family	ID	Variant	Zygosity	Sex	Age (years)	Height (cm)	Height Standard Deviation	Head Circumfer-ence (cm)	Head circumfer-ence Standard deviation	Genu valgum
ZFD-01	III:1	*PCNT* c.6176_6189del GTCAGCTGCCGAAG	Heterozygous	F	45	155	-1.2	NA	NA	-
	III:2		Heterozygous	M	54	161	-2.1	NA	NA	-
	IV:1		Homozygous	M	27	117	-8.2	45	-6.8	-
	IV:3		Homozygous	M	20	123	-7.3	47	-5.4	-
	IV:5		Wild type	F	12	155	0.6	54	+ 0.7	-
ZFD-02	III:2	*RAB33B* c.174delC	Heterozygous	F	49	150	-2.0	NA	NA	-
	III:3		Heterozygous	M	54	160	-0.4	NA	NA	-
	III:4		Heterozygous	F	45	150	-1.9	NA	NA	-
	IV:1		Heterozygous	M	35	159	-2.4	NA	NA	-
	IV:2		Homozygous	F	33	112	-7.2	49	-5.0	+
	IV:3		Homozygous	F	29	117	-6.5	53	-1.2	+
	IV:8		Homozygous	F	22	122	-5.7	51	-3.1	-
	IV:10		Heterozygous	M	7	122	0	54	+ 1.4	-
	IV:11		Homozygous	M	10	104	-4.9	48	-3.7	+
	IV:12		Wild type	F	13	143.5	-1.8	58	+ 3.4	-

The affected individuals of family ZFD-02 had disproportionately short statures (Fig. 1G). Individuals IV:2, IV:3, IV:8 and IV:11 were of ages 33, 29, 22 and 10 years respectively (Table 1). The symptoms became evident after the age of 2 years of age. They had restricted joint mobility and valgus deformity in knee joints and barrel shaped chest (Fig. 1H-J). As subjects aged, the disorder grew progressively with movement becoming more difficult. The oldest affected individual had a severely affected and painful gait. Severe platyspondyly, genu valgus deformity of legs, cupping of distal ribs, pectus carinatum, and irregular iliac crests were observed in radiographs of individual IV:11 (Fig. 1K-N).

### Molecular Analysis

Whole genome sequencing of the affected individual IV:1 of family ZFD-01 revealed eight variants (Table S1) after analysis and filtration of data. Out of these, only a deletion of 14 bp, c.6176_6189del GTCAGCTGCCGAAG; p.(Gln2060ArgfsTer48) in exon 30 of *PCNT* (NM_001315529.2) (Fig. 1O) segregated with the disorder. This finding was consistent with a diagnosis of MOPDII for the patients.

In family ZFD-02, whole genome sequencing of the affected individual IV:3 revealed four variants after filtration by applying different criteria (Table S2). A frameshift variant in *RAB33B* (NM_031296.1), c.174delC; p.(Asp60ThrfsTer7) segregated fully with the phenotype (Fig. 1P) and confirmed the diagnosis of SMC2 (OMIM 615,222). This variant was absent in all public databases. The *RAB33B* (NM_031296.1), c.174delC; p.(Asp60ThrfsTer7) variant has been deposited in LOVD with ID 0,000,670,684.

The *PCNT* gene is comprised of 47 coding exons which encodes a 3,336 amino acids long coiled coil protein flanked at terminals by non-helical regions. The mutation identified in family ZFD-01 in *PCNT* gene is presumed to produce a non-functional truncated protein consisting of 2108 amino acids, if the mRNA escapes nonsense mediated decay. Similarly, the mutation identified in family ZFD-02 in *RAB33B* gene will severely truncate the protein to 67 amino acids only, if the mRNA is translated. However, both mutant transcripts of *PCNT* and *RAB33B* are likely to undergo nonsense-mediated decay and no proteins will be produced.

## Discussion

In this study we used whole genome sequencing of the index subjects in two families with rare forms of skeletal dysplasia to identify the disease-causing gene defects. In family ZFD-01 we identified a homozygous frameshift variant in *PCNT*, confirming the diagnosis of MOPDII. The affected individuals had severe but proportionate short stature (heights SD < -6.8). Their head circumference was also reduced as observed in previously described patients with MOPDII. Around 50% of affected individuals with MOPDII develop cerebral neurovascular abnormalities [7]. Dentition problems are also common. However, in our patients, cognition was not impaired and both affected individuals had normal dentition.

*PCNT* is located on chromosome 21 and encodes pericentrin, a centrosomal protein responsible for nucleation of mitotic spindle and binding to calmodulin [8]. It networks with its other components including the γ-tubulin ring complex and provides an active configuration for the proteins, essential for the Microtubular organizing center activities. Disruptions in astral microtubule and spindle orientation can lead to the malfunctioning of Pericentriolar Matrix, which plays a crucial role in organization of spindles and can lead to mitotic arrest or cell death [8, 9].

Various pathogenic variants of *PCNT* have been identified in MOPDII and Seckel syndrome [1] (www.hgmd.cf.ac.uk accessed June 2021). The 14 nucleotide deletion, c.6176_6189 del15 in exon 30, revealed by analysis of the whole genome sequencing data in family ZFD-01, has been previously reported in a Pakistani female patient with similar phenotypic features. She had many skeletal manifestations as described for MOPDII as well as polycystic ovaries [1].

In the second family ZFD-02 a novel homozygous variant c.174delC, p.(Asp60ThrfsTer7) was identified in *RAB33B*. The affected individuals had typical features of Smith–McCort dysplasia (SMC). Skeletal features on radiographs, including severe platyspondyly, short and broad metacarpals, abnormal carpal bones and lower limb valgus deformity were in concordance with the previously reported cases of SMC phenotypes [4, 5]. These also included barrel chests, limited joint movement, lower limb deformities and normal intellect.

*RAB33B* (NM_031296.2) is located on chromosome 4q31.1, comprises 2 exons and encodes RAB33B which is localized to Golgi complex [3]. RAB33B is a type of Rab GTPase. Rab GTPase membrane bound proteins are involved in membrane transport. It is involved in retrograde Golgi transport (from Golgi to ER) of proteins [10]. Moreover, it also functions in the formation of autophagosomes by binding with the Atg12-5/16L complex, contributing to macroautophagy [4].

To date, only seven variants of *RAB33B* have been identified in patients with a similar phenotype, all from different ethnic backgrounds [3–5] (www.hgmd.cf.ac.uk accessed June 2021). A missense mutation in *RAB33B* can cause marked deficiency of RABB33B inside Golgi and affects the vesicular trafficking of Golgi complex [4]. This novel variant c.174delC p.(Asp60ThrfsTer7) identified in family ZFD-02, will most likely mark the mRNA for nonsense mediated decay, or if the mutant transcript escapes nonsense mediated decay, premature truncation of the protein within the GTPase domain will occur (Fig. 1Q).

## Conclusion

The findings of this present study suggest phenotypic homogeneity of patients harboring *PCNT* or *RAB33B* variants. The c.174delC; p.(Asp60ThrfsTer7) variant identified in this research adds to the allelic spectrum of *RAB33B* variants of this rare genetic disorder. Our study further shows that for genetic diagnosis of rare recessive skeletal dysplasias, whole genome sequencing of the index case provides a suitable diagnostic approach.

## Supplementary Information

**Additional file 1.**

## Data Availability

Data generated in this study is available upon reasonable request to the corresponding authors. We have deposited the sequence variant data in LOVD (ID 0,000,670,684). Due to ethical concerns, our IRB has not approved the deposition of whole genome sequencing or exome sequencing data in a public database.
