# Supplementary material for: RAB33B and PCNT variants in two Pakistani families with skeletal dysplasia and short stature
Source: BMC Musculoskelet Disord. 2021 Jul 20;22:630. doi: 10.1186/s12891-021-04503-2 (PMC8293541; doi:10.1186/s12891-021-04503-2)
Supplement: Supplementary file 1 — Additional file 1. [file 12891_2021_4503_MOESM1_ESM.docx]

***RAB33B* and *PCNT* variants in two Pakistani families with skeletal dysplasia and short stature**

Noor ul Ain^1,2^, Zunaira Fatima^1^, Sadaf Naz^1*^, Outi Makitie^2,3,4*^

^1^School of Biological Sciences, University of the Punjab, Lahore, Pakistan

^2^Department of Molecular Medicine and Surgery and Center for Molecular Medicine, Karolinska Institutet, Stockholm, Sweden

^3^ Folkhälsan Institute of Genetics, Helsinki, Finland

^4^ Children's Hospital, University of Helsinki and Helsinki University Hospital, Helsinki, Finland

NA current address Institute of Biomedical and Genetic Engineering, Islamabad, Pakistan

SN and OM, equal contribution

***Corresponding authors:**

Sadaf Naz

School of Biological Sciences, University of the Punjab, Quaid-i-Azam Campus, Lahore 54590, Pakistan. Tel: +92-42-99231819, email: naz.sbs@pu.edu.pk

Outi Makitie

P.O. Box 63, FI-00014 University of Helsinki, Helsinki, Finland

outi.makitie@helsinki.fi

**Supplementary Material**

**Table S1:** Filtered variants identified in family ZFD-01 after analysis of WGS data.

| **Chr** | **Start*** | **Reference** | **Change** | **Gene** | **Transcript** | **gnomAD Freq** |
| --- | --- | --- | --- | --- | --- | --- |
| 4 | 191002356 | A | T | *DUX4L4* | ENST00000538692 | 0 |
| 4 | 191002467 | T | C | *DUX4L4* | ENST00000538692 | 0 |
| 14 | 92537353 | C | CCTGCTGCTGCTGCTGCTGCTGCTGCTG | *ATXN3* | ENST00000340660 | 0 |
| 1 | 13368548 | C | T | *PRAMEF5* | ENST00000376168 | 0 |
| 9 | 133556991 | T | TCGC | *PRDM12* | ENST00000253008 | 0 |
| 22 | 51135983 | G | GGCCCCGGCCCCGCGCCCGGC | *SHANK3* | ENST00000262795 | 0 |
| 21 | 47836006 | TGTCAGCTGCCGAAG | T | *PCNT* | ENST00000359568 | 0.00000398 |
| 3 | 195453046 | GTTAGCA | G | *MUC20* | ENST00000320736 | 0 |

*Positions are with reference to GRCh37/hg19.

**Table S2:** Filtered variants identified in family ZFD-02 after analysis of WGS data

| **Chr** | **Start*** | **Reference** | **Change** | **Gene** | **Transcript** | **gnomAD Freq** |
| --- | --- | --- | --- | --- | --- | --- |
| 4 | 140375521 | TC | T | *RAB33B* | ENST00000305626 | 0 |
| 22 | 51135967 | CT | C | *SHANK3* | ENST00000262795 | 0 |
| 22 | 51135983 | G | GGCCCCGGCCCCGCGCCCGGC | *SHANK3* | ENST00000262795 | 0 |
| 17 | 71354264 | G | A | *SDK2* | ENST00000388726 | 0.00006031 |

*Positions are with reference to GRCh37/hg19.
